# *Schistosoma*-associated *Salmonella *resist antibiotics via specific fimbrial attachments to the flatworm

**DOI:** 10.1186/1756-3305-4-123

**Published:** 2011-06-28

**Authors:** Alison E Barnhill, Ekaterina Novozhilova, Tim A Day, Steve A Carlson

**Affiliations:** 1 Department of Biomedical Sciences, College of Veterinary Medicine, Iowa State University, 1600 SE 16th St., Ames, IA 50011, USA

## Abstract

**Background:**

Schistosomes are parasitic helminths that infect humans through dermo-invasion while in contaminated water. *Salmonella *are also a common water-borne human pathogen that infects the gastrointestinal tract via the oral route. Both pathogens eventually enter the systemic circulation as part of their respective disease processes. Concurrent *Schistosoma-Salmonella *infections are common and are complicated by the bacteria adhering to adult schistosomes present in the mesenteric vasculature. This interaction provides a refuge in which the bacterium can putatively evade antibiotic therapy and anthelmintic monotherapy can lead to a massive release of occult *Salmonella*.

**Results:**

Using a novel antibiotic protection assay, our results reveal that *Schistosoma*-associated *Salmonella *are refractory to eight different antibiotics commonly used to treat salmonellosis. The efficacy of these antibiotics was decreased by a factor of 4 to 16 due to this association. *Salmonella *binding to schistosomes occurs via a specific fimbrial protein (FimH) present on the surface on the bacterium. This same fimbrial protein confers the ability of *Salmonella *to bind to mammalian cells.

**Conclusions:**

*Salmonella *can evade certain antibiotics by binding to *Schistosoma*. As a result, effective bactericidal concentrations of antibiotics are unfortunately above the achievable therapeutic levels of the drugs in co-infected individuals. *Salmonella-Schistosoma *binding is analogous to the adherence of *Salmonella *to cells lining the mammalian intestine. Perturbing this binding is the key to eliminating *Salmonella *that complicate schistosomiasis.

## Background

Schistosomes are parasitic helminths that infect humans, with life cycles involving snails as intermediate hosts. Schistosomiasis occurs in 74 developing tropical and subtropical countries in which over 200 million people are infected. Of those, 120 million patients show symptoms with 20 million severely infected. There are 14,000 deaths per year due to schistosomiasis [1].

*Salmonella *spp. is a common water- and food-borne cause of gastrointestinal and systemic diseases worldwide. Approximately 2 million individuals die each year from diarrheal disease and *Salmonella *is a leading cause of this malady. In the U.S. alone, *Salmonella *causes about 1.4 million infections per year [2].

Concurrent *Schistosoma-Salmonella *infections occur when enteroinvasive *Salmonella *enter the systemic circulation and attach to the tegument of adult *Schistosoma *[3,4] present in the mesenteric vasculature. This interaction apparently provides a refuge in which the bacterium can evade systemic antibiotic therapy. For example, chloramphenicol-sensitive *S. typhi *were demonstrated to be refractory to chloramphenicol treatment in co-infections [5]. Furthermore, therapy with the anthelmintic praziquantel can lead to a massive release of schistosome-associated *Salmonella *causing peracute septicemia if the appropriate antibiotic is not co-administered to co-infected children [6,7]. Finally, the use of ineffective antibiotics contributes to antibiotic resistance development and the phenomenon of bacterial persistence.

To assess the nature and extent of this phenomenon, we developed an *in vitro *antibiotic protection assay in which anti-*Salmonella *antibiotic efficacy is evaluated using various *Salmonella *incubated with adult schistosomes and a variety of antibiotics. These studies identify *Salmonella *factors that facilitate attachment to *Schistosoma *while also cataloguing the antibiotics that are ineffective against *Salmonella *adhering to schistosomes.

## Results

### Assessment of qualitative amoxicillin resistance in *Salmonella *adhering to schistosomes

To determine if *Salmonella *can evade the effects of antibiotics through adherence to schistosomes, we developed a novel antibiotic protection assay in which antibiotic-sensitive *Salmonella typhimurium *(strains listed in Table 1) were incubated with a therapeutically-relevant concentration of amoxicillin (32 μg/ml) known to be 100% lethal to these strains in the absence of schistosomes [2]. *Salmonella typhimurium *strains included SL1344 (invasive), BJ68 (non-invasive), and EE419 (hyperinvasive). As part of the control conditions, bacterial killing was determined prior to co-incubation with schistosomes and following adherence to schistosomes. Additional controls included assessing amoxicillin-mediated killing of *Salmonella *adhering to *Girardia tigrina *(free-living flatworms) and to HEp-2 mammalian tissue culture cells. A laboratory strain of *E. coli *(Top10) was also used as a control.

**Table 1:** Summary of bacteria used in this study

Bacterium	Relevant Characteristics	Reference
*Salmonella typhimurium *strain SL1344	Invasive; adherent to mammalian cells	[12]

*Salmonella typhimurium *strain BJ68	Non-invasive; adherent to mammalian cells	[13]

*Salmonella typhimurium *strain EE419	Hyperinvasive; adherent to mammalian cells	[14]

*Salmonella paratyphi*	Adapted to humans; adherent to mammalian cells	[9]

*Salmonella pullorum/gallinarum*	Adherent to avian cells	[8]

*Salmonella pullorum*/pFimH	Engineered to be adherent to mammalian cells via heterologous expression of FimH from SL1344	This study

*Salmonella typhimurium*/dFimH	Engineered to be non-adherent to mammalian cells via transposon-mediated deletion of *fimH*	This study

*Salmonella javiana*	Adherent to mammalian and reptilian cells	[15]

*Shigella flexneri*	Human pathogen related to *Salmonella *	[19]

*E. coli *Top10	Non-invasive and non-adherent	

*E. coli *(ETEC, EPEC, and EHEC)	Invasive and adherent	

**Figure 1:**
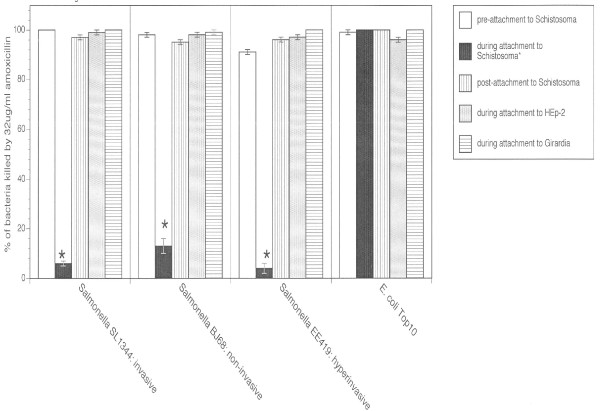
**Amoxicillin resistance in *Salmonella *adhering to schistosomes (black boxes)**. Approximately 10^4 ^CFUs of antibiotic-sensitive *Salmonella typhimurium *were incubated with a single adult *Schistosoma *for two hours followed by the addition of the "breakpoint" concentration [16] of amoxicillin (32 μg/ml). Following washing of the antibiotic, *Salmonella *were liberated from eukaryotes by trypsinization and bacteria were then recovered and enumerated for determination of percent bacterial killing. *Salmonella typhimurium *strains include SL1344 (invasive), BJ68 (non-invasive), and EE419 (hyperinvasive). Control conditions include determining bacterial killing prior to co-incubation with schistosomes (open boxes) and following adherence to schistosomes (vertical hatching). Additional controls included the assessment of antibiotic-mediated killing of *Salmonella *adhering to *Girardia *free-living flatworms (horizontal hatching) and HEp-2 mammalian tissue culture cells (gray boxes). A laboratory strain of *E. coli *(Top10) was also used as a control. Data presented are the mean ± SEM for three independent experiments each performed in triplicate. *, bacterial survival was significantly higher (p <0.01) during adherence to schistosomes when compared to the other conditions for *Salmonella*.

As shown in Figure 1, bacterial survival was significantly higher (p <0.01) during adherence to schistosomes when compared to the other conditions for *Salmonella*. Specifically, whereas 32 μg/ml amoxicillin kills essentially 100% of the *Salmonella *strains incubated alone, it kills less than 12% of the same strains incubated with adult schistosomes. This schistosome protective effect was not dependent upon the ability of *Salmonella *to invade the eukaryotic cells since the non-invasive strain was protected from amoxicillin in a manner similar to that observed for invasive and hyperinvasive strains. The protective effect could not be replicated by co-incubation with the free-living flatworm *G. tigrina *or with HEp-2 mammalian tissue culture cells. Further, *S. mansoni *afforded no protection to *E. coli*.

### Quantitative evaluation of amoxicillin resistance in *Salmonella *adhering to schistosomes

To assess the concentration-dependent nature of schistosome-mediated *Salmonella *resistance to antibiotic killing, we used the antibiotic protection assay to obtain amoxicillin kill curves for *Salmonella *adhering to schistosomes. *Salmonella *and *Schistosoma *were co-incubated in the presence of various concentrations of amoxicillin. Control conditions include determining bacterial killing in the presence of HEp-2 mammalian tissue culture cells.

**Figure 2:**
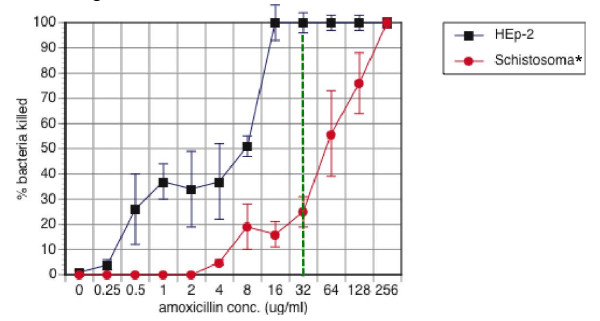
**Kill-curve obtained for *Salmonella *incubated with *Schistosoma *or HEp-2 cells in the presence of various concentrations of amoxicillin**. Approximately 10^4 ^CFUs of antibiotic-sensitive *Salmonella typhimurium *were incubated with a single adult *Schistosoma *or HEp-2 cells for two hours followed by the addition of various concentrations of amoxicillin. Data presented are the percentages of *Salmonella *killed by amoxicillin during attachment to schistosomes (black circles) or HEp-2 cells (red boxes). The native breakpoint for amoxicillin (32 μg/ml [17]) is indicated by the vertical dashed green line. Data presented are the mean ± SEM for three independent experiments each performed in triplicate. *p <0.01 versus HEp-2 controls for 0.5-128 μg/ml of amoxicillin. Similar results were observed for other antibiotics (summarized in Table 2).

For most concentrations of amoxicillin, bacterial survival was significantly higher (p <0.01) during adherence to schistosomes when compared to HEp-2 cells adherence (Figure 2). The apparent minimum inhibitory concentration (MIC), *i.e*. the lowest concentrations that kills or inhibits the growth of 100% of *Salmonella *in an *in vitro *inoculum containing 10^6 ^bacteria/ml, was 16 μg/ml in the presence of HEp-2 cells but 16 times higher for *Salmonella *adhering to *Schistosoma*.

### Evaluation of *Schistosoma*-mediated protection of *Salmonella *from other antibiotics

To determine if *Salmonella *can evade the effects of antibiotics other than amoxicillin, studies presented in Figure 1 were repeated using the invasive strain of *Salmonella *(SL1344) and clinically-relevant concentrations of seven other antibiotics. Other antibiotics included cefepime, cefpodoxime, chloramphenicol, ciprofloxacin, streptomycin, sulfadimethoxine, or tetracycline. Cefepime (4^th ^generation cephalosporin), cefpodoxime (3^rd ^generation cephalosporin), and ciprofloxacin (fluoroquinolone) represent the antibiotics currently recommended for use against systemic salmonellosis [2]. Chloramphenicol was chosen because of documented treatment failures in *Salmonella-Schistosoma *co-infections despite chloramphenicol sensitivity exhibited by the *Salmonella *isolate [5]. Streptomycin, sulfadimethoxine, and tetracycline are other antibiotics that have been previously used to treat systemic salmonellosis [2].

**Figure 3:**
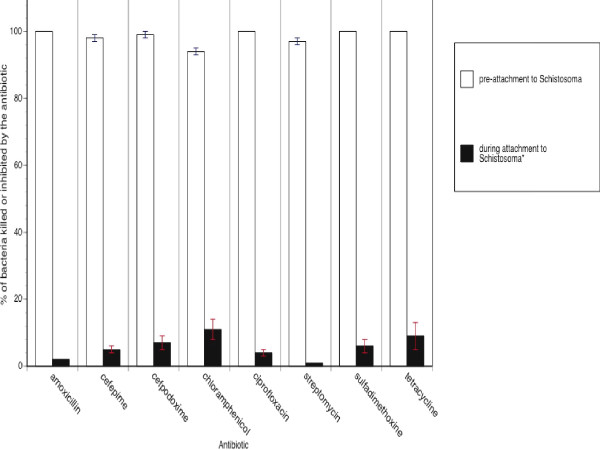
***Schistosoma*-mediated protection of *Salmonella *exposed to amoxicillin, cefepime, cefpodoxime, chloramphenicol, ciprofloxacin, streptomycin, sulfadimethoxine, or tetracycline**. Approximately 10^4 ^CFUs of antibiotic-sensitive *Salmonella typhimurium *were incubated with a single adult *Schistosoma *for two hours followed by the addition of the "breakpoint" concentration [17] of an antibiotic. Data presented are the percentages of *Salmonella *killed or inhibited by antibiotics prior to incubation with schistosomes (open boxes) or in the presence of *Schistosoma *(black boxes). Data presented are the mean ± SEM for three independent experiments each performed in triplicate. Bacterial survival was significantly higher (p <0.01) during adherence to schistosomes when compared to the schistosome-free condition for a given antibiotic. No significant differences were noted for survival of bacteria prior to schistosome attachment, following attachment to schistosomes, in the presence of HEp-2 cells, or in the presence of *Girardia *(data not shown).

As shown in Figure 3, bacterial survival was significantly higher (p <0.01) during adherence to schistosomes when compared to the other conditions for all antibiotics. Table 2 illustrates the quantitative nature of the resistance where adherence to *Schistosoma *significantly increased the apparent MIC values beyond the clinical breakpoint, *i.e*. MIC value that is ascribed as the threshold for sensitivity/resistance, for each antibiotic. Again, this protection was not provided by co-incubation with either *G. tigrina *or mammalian cells, where in every instance killing was not significantly lower than 100%. Also, this protection was not afforded to *E. coli *incubated with schistosomes.

**Table 2:** Apparent MIC data for *Salmonella *adhering to *Schistosoma*.

Antibiotic	MICs and Breakpoints			
	
	MIC prior to exposure to *Schistosoma*	MIC during adherence to *Schistosoma*	MIC following exposure to *Schistosoma*	**Breakpoint of the antibiotic for *Salmonella ***[17]
amoxicillin	16 μg/ml	*256 μg/ml*	16 μg/ml	32 μg/ml

cefepime	16 μg/ml	*128 μg/ml*	16 μg/ml	32 μg/ml

cefpodoxime	32 μg/ml	*256 μg/ml*	32 μg/ml	32 μg/ml

chloramphenicol	16 μg/ml	*64 μg/ml*	16 μg/ml	32 μg/ml

ciprofloxacin	0.25 μg/ml	*8 μg/ml*	0.25 μg/ml	4 μg/ml

streptomycin	16 μg/ml	*256 μg/ml*	16 μg/ml	32 μg/ml

sulfadimethoxine	128 μg/ml	*1024 μg/ml*	64 μg/ml	512 μg/ml

tetracycline	8 μg/ml	*64 μg/ml*	8 μg/ml	16 μg/ml

Italicized number indicate resistance to the respective antibiotic. Adherence to HEp-2 cells or *Girardia *had no effect on the apparent MIC values for *Salmonella *(not shown). Adherence to schistosomes had no effect on the apparent MIC values for pathogenic and avirulent *E. coli *(not shown).

### Assessment of FimH as a determinant of *Salmonella *adherence to *Schistosoma*

Previous studies indicated that *Salmonella *fimbrial proteins participate in the binding to *Schistosoma *[3,4]. Fimbrial proteins, specifically FimH, confer selective binding properties to mammalian versus avian hosts. The T78⇒I mutation in FimH confers avian cell binding to *Salmonella pullorum *and *S. gallinarum *[8] whereas the wild-type FimH confers mammalian cell binding to many *Salmonella *including *S. typhimurium *and the human-adapted *S. paratyphi*. Therefore, this mutation may play a role in *Salmonella-Schistosoma *adherence. In the present study, schistosome-mediated protection from antibiotics was evaluated in *Salmonella *expressing mammalian cell-binding FimH (threonine at amino acid 78) or avian cell-binding FimH (isoleucine at amino acid 78).

**Figure 4:**
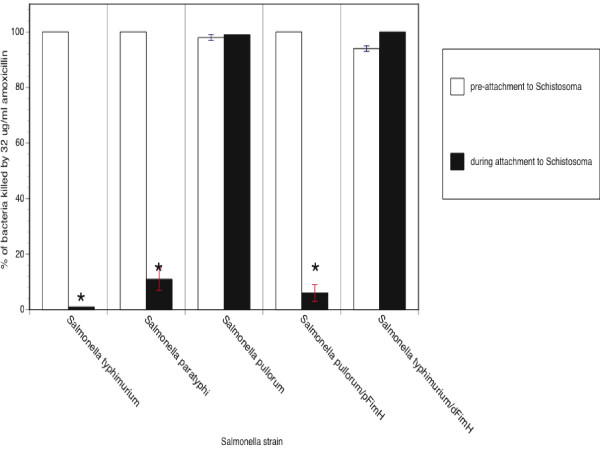
**Amoxicillin killing of various *Salmonella *strains adhering to schistosomes**. *Salmonella *and *Schistosoma *were co-incubated, exposed to 32 μg/ml of amoxicillin, recovered and enumerated as described above. Data presented are the percentages of *Salmonella *killed or inhibited by antibiotics prior to incubation with schistosomes (open boxes) or in the presence of *Schistosoma *(black boxes). Bacterial survival was significantly higher (p <0.01) during adherence to schistosomes for *Salmonella *expressing the FimH that confers mammalian cell adherence. No significant differences were noted for survival of bacteria prior to schistosome attachment, following attachment to schistosomes, in the presence of HEp-2 cells, or in the presence of *Girardia *(data not shown).

As shown in Figure 4, protection from antibiotics was only afforded to *Salmonella *expressing mammalian cell-binding FimH. Deletion of FimH abrogated the schistosome-mediated protection for *Salmonella typhimurium*. Further, *Salmonella pullorum*, which was not protected by co-incubation with schistosomes, became protected with the heterologous expression of the mammalian cell-binding FimH. Table 3 summarizes the relationship between expression of mammalian cell binding FimH and the antibiotic protection afforded by schistosomes.

**Table 3:** Summary of antibiotic resistance in bacteria associated with *Schistosoma*.

Bacteria (all natively sensitive to antibiotics)	Relevant Characteristics	Resistant or Sensitive
*Salmonella typhimurium*; *Salmonella paratyphi*; *Salmonella pullorum*/pFimH; *Salmonella javiana*	Express a FimH protein with Thr at amino acid 78	Resistant

*Salmonella typhimurium*/dFimH; *Salmonella pullorum*; *Salmonella gallinarum*	Express a FimH protein with Ile at amino acid 78	Sensitive

*Shigella flexneri*; *E. coli *	Invade and attach via alternative processes	Sensitive

Resistance/sensitivity determinations are based on apparent MIC values exceeding the breakpoint for each of the eight antibiotics examined.

## Discussion

A previous study documents antibacterial treatment failures in *Schistosoma-Salmonella *co-infections despite *in vitro *sensitivity to the drug in the absence of schistosomes [5]. Other studies revealed that *Salmonella *can adhere to the tegument of *Schistosoma *and that this adherence is dependent upon fimbriae present on the surface of *Salmonella *[3,4]. The study herein describes experiments addressing the hypothesis that *Schistosoma*-associated *Salmonella *can evade a number of antibiotics by binding to adult schistosomes using fimbrial proteins implicated in mammalian cell adherence.

Antibiotic protection studies revealed that eight different anti-*Salmonella *antibiotics were incapable of effectively killing or inhibiting the bacterium when adhered to *Schistosoma*, despite efficacy of these drugs against *Salmonella *that were not associated with schistosomes. This effect was not related to *Salmonella *invading the tegumental cells of schistosomes since a non-invasive isostrain was recalcitrant to the antibiotics and a hyperinvasive isostrain did not exhibit any significant elevation of antibiotic resistance. This phenomenon was not observed for *Salmonella *binding to other eukaryotic cells such as HEp-2 mammalian epithelial cells or tegumental cells on the surface of the free-living flatworm *G. tigrina*. Bacterial growth was minimal and equivalent for *Salmonella *adhering to schistosomes, HEp-2 cells, or when incubated with only RPMI media (data not shown).

The binding of *Salmonella *to *Schistosoma *is dependent upon a specific fimbrial protein expressed by *Salmonella *that attach to mammalian cells. Various *Salmonella *serotypes have specific host tropisms and fimbrial proteins dictate this host range phenomenon [8]. *Salmonella pullorum *and *Salmonella gallinarum *are avian-adapted *Salmonella *and schistosomes protected neither of these serotypes whereas broad host range serotypes, like *S. typhimurium *and *S. javiana*, were protected from the antibiotics. Mammalian-adapted *Salmonella paratyphi*, which is adapted to humans [9], is refractory to the antibiotics upon association with *Schistosoma*. FimH is a fimbrial protein that endows selective eukaryotic cell adherence [8] and our studies revealed that schistosomal adherence is absent for *Salmonella *that express the avian cell-specific version of this fimbria. It would therefore appear that *Schistosoma *bear a cell surface epitope that is analogous to the mammalian cell-docking site for FimH.

Since the antibiotic resistance is transient and only occurs during adherence, the mechanism for the resistance is likely a physical barrier that may include a biofilm. The glycocalyx of *Schistosoma *is thick, highly immunogenic, and fucose-rich [10] suggesting that antibiotics may poorly penetrate this milieu. Given the chemical diversity of the antibiotics used in this study, it is unlikely that the schistosome glycocalyx is capable of chemically altering and inactivating the drugs. Bacterial quiescence is another possibility but *Salmonella *depend on folate auto-deprivation and the temporal nature of this phenomenon [11] is not likely to occur during the protection assay. The same can be said for the activation of other metabolic changes in *Salmonella *physiology, *i.e*., the two hour incubation period is likely insufficient for inducing resistance. Further studies will address the physical basis for the phenomenon described herein.

## Conclusions

*Schistosoma*-associated *Salmonella *can evade certain antibiotics during adherence to the flatworm. As a result, traditional anti-*Salmonella *drugs are not useful in co-infected individuals. Bacteria-flatworm binding mimics the adherence of *Salmonella *to cells lining the mammalian intestine. Perturbing this binding is the key to eliminating *Salmonella *that complicate schistosomiasis.

## Methods

### Summary of microbes used in this study

Bacterial strains are summarized in Table 1 with *Salmonella typhimurium *strain SL1344 [12] serving as the model invasive strain. Non-invasive isostrain BJ68 [13] and hyperinvasive strain EE419 [14] are both derivatives of SL1344. Other *Salmonella *include human-adapted *S. paratyphi *[9] and the broad host-range *S. javiana *that infects mammals and reptiles [15]. Avian-adapted *S. pullorum *was also used in these studies. Bacteria were stored in cryopreservation tubes containing 50% glycerol:50% culture medium at -80°C and grown in LB broth (Sigma) without antibiotics.

To assess the role of specific fimbriae in the adherence of *Salmonella *to schistosomes, the *fimH *gene was PCR-amplified from SL1344 and cloned into the prokaryotic expression vector pCR2.1 (Invitrogen). This plasmid, designated as pFimH, was transformed into *Salmonella pullorum *and the transformant is designated as *Salmonella pullorum*/pFimH.

Additionally, *S. typhimurium *was engineered to express the *S. pullorum*-specific FimH bearing the T78⇒I mutation that abrogates adherence to mammalian cells. First, the *TnZeo *[16] transposon was inserted into *fimH *of SL1344 thus preventing expression of native *fimH*. Since this deletion will have polar effects on the polycistronic *fim *transcript, the *fim *operon (*fimABCDHFZYW*) was PCR-amplified from *S. pullorum*, cloned into the pCRXL prokaryotic expression vector, and transformed into SL1344 bearing the *TnZeo *insertion into *fimH*. This strain is designated as *Salmonella typhimurium*/dFimH.

Non-*Salmonella *bacteria included pathogenic *Shigella*, pathogenic *E. coli*, and a laboratory strain of *E. coli*. These bacteria are related to *Salmonella *but they express fimbriae that are divergent from those found in *Salmonella*.

### Isolation of parasites

Adult male *Schistosoma mansoni *worms were recovered 45-60 days post-infection from portal and mesenteric veins of Swiss Webster female mice provided by the Biomedical Research Institute, Rockville MD, USA. All animal procedures were conducted in accordance with Iowa State University's approved animal care protocol #07-I-025-A/H.

### Antibiotic protection assay

Approximately 10^4 ^CFUs of antibiotic-sensitive bacteria were incubated with a single adult *Schistosoma *in 12-well tissue culture dishes containing 1 mL of RPMI (Invitrogen) with 50% fetal bovine serum (Difco). Co-incubations lasted for two hours followed by the addition of the "breakpoint" concentrations [17] of one of the following antibiotics: amoxicillin (32 μg/ml), cefepime (32 μg/ml), cefpodoxime (32 μg/ml), chloramphenicol (32 μg/ml), ciprofloxacin (4 μg/ml), streptomycin (32 μg/ml), sulfadimethoxine (512 μg/ml), or tetracycline (16 μg/ml). All antibiotics were obtained from Sigma Chemicals.

Following washing of the antibiotic after 2 hrs, trypsin (0.05%) was then added in order to cleave the *Salmonella *fimbriae that mediate the attachment to *Schistosoma *[3,18]. Media was then removed and plated on *Salmonella*-selective agar that was incubated at 37°C overnight. *Salmonella *colonies were then enumerated for determination of percent of bacteria recovered, *i.e*. an indirect measurement of bacterial killing. Percent bacterial killing equals 100 ×(10^4 ^minus the number of CFUs recovered)/10^4^.

Control conditions included determining bacterial killing prior to co-incubation with schistosomes, during adherence to schistosomes, and following adherence to schistosomes. The latter situation refers to worms that were removed from the schistosomes and then examined for antibiotic susceptibilities, a measurement of the duration (or lack thereof) of the resistance afforded by schistosomes. Additional controls included the assessment of antibiotic-mediated killing of *Salmonella *adhering to *Girardia *free-living flatworms and HEp-2 mammalian tissue culture cells.

### Concentration-response analysis of antibiotic resistance exhibited by *Schistosoma*-associated *Salmonella typhimurium *SL1344

Various concentrations of antibiotics (0-1,024 μg/ml) were used in the antibiotic protection assay described above. Percent bacterial killing was determined for SL1344 adhering to schistosomes, *Girardia*, or HEp-2 cells. As a control, bacterial killing was assessed in eukaryotic cell-free assays and in the presence of HEp-2 cells. Apparent antibiotic minimum inhibitory concentrations (MICs) were ascribed to the lowest concentration of antibiotic that inhibited or killed 100% of the bacterial inocula.

### Statistics

Statistical analysis was performed using ANOVA with Scheffe's F test for multiple comparisons. Comparisons were made between strains and between incubation conditions.

## Competing interests

The authors declare that they have no competing interests.

## Authors' contributions

TAD and SAC conceived and designed the experiments and analyzed the data; AEB, EN, and SAC performed the experiments. Manuscript was primarily written by SAC with assistance from the other three co-authors. All authors read and approved the final manuscript.
